# Noninvasive Follicular Thyroid Neoplasm with Papillary-Like Nuclear Features in Asian Practice: Perspectives for Surgical Pathology and Cytopathology

**DOI:** 10.1007/s12022-018-9519-6

**Published:** 2018-02-23

**Authors:** Andrey Bychkov, Chan Kwon Jung, Zhiyan Liu, Kennichi Kakudo

**Affiliations:** 1 Department of Pathology, Faculty of Medicine, Chulalongkorn University, Bangkok, 10330 Thailand; 2 Department of Hospital Pathology, College of Medicine, The Catholic University of Korea, Seoul, 06591 South Korea; 3 Department of Pathology, Shandong University School of Basic Medical Sciences, Jinan, Shandong 250012 China; 4 Department of Pathology, Nara Hospital, Kindai University Faculty of Medicine, Nara, 630-0293 Japan

**Keywords:** NIFTP, Asia, Thyroid cancer, Papillary thyroid carcinoma, Follicular variant of papillary thyroid carcinoma

## Abstract

The introduction of noninvasive follicular thyroid neoplasm with papillary-like nuclear features (NIFTP) was initiated and promoted by pathologists. Recent Asian studies added new knowledge to the existing literature to aid a better understanding of NIFTP. Our original data and the results of a meta-analysis suggest that the initial rate of NIFTP has been overestimated, averaging 9.1% (95% confidence interval [CI] 6.0–12.7%) of all papillary thyroid cancers worldwide. The incidence of NIFTP in the Asian population (1.6%, 95% CI 0.9–2.5%; 7 studies) is significantly lower than that reported in the non-Asian series (13.3%, 95% CI 9.0–18.3%; 18 studies). Such difference could be attributed to various perceptions of histological diagnostic thresholds, different nature of papillary thyroid carcinoma, and different approaches in the management of thyroid nodules. The active surveillance for indeterminate nodules and NIFTP, largely represented in the indeterminate cytologic categories, promoted by Japanese institutions establishes a new paradigm to reduce overtreatment of these patients. The lower prevalence of NIFTP in the Asian series indicates a low impact on the risk of malignancy in cytopathology, as it was demonstrated in our original multi-institutional cohort of thyroid nodules, and may predict a low impact on the performance of commercial molecular tests. Several Korean studies addressed the issue of *BRAF* mutation in NIFTP, which prompted the current refinement of the diagnostic criteria for NIFTP. Our survey of Asian pathologists found that the term NIFTP has not been universally adopted in the local practice. Endocrine pathologists must promote the new entity through provision of educational activities.

The introduction of noninvasive follicular thyroid neoplasm with papillary-like nuclear features (NIFTP) to replace the noninvasive encapsulated follicular variant of papillary thyroid carcinoma (eFV-PTC) was initiated and promoted by pathologists [1]. Presently, after 1.5 years of adopting a new terminology, NIFTP remains the topic of interest in the field, echoed in numerous publications authored by thyroid pathologists and cytopathologists. However, the impact of this reclassification extends beyond the field of pathology. Previous studies proposed that the introduction of NIFTP has significant implications for patients and clinicians. In fact, NIFTP was primarily addressed to reduce the overdiagnosis and overtreatment of indolent thyroid cancers, that is, noninvasive eFV-PTC [1]. Management strategies and guidelines have been adjusted to adopt the new terminology [2, 3]. As a result, patients with NIFTP are no longer treated aggressively and not labeled as cancer patients. Finally, the health care system may expect significant cost savings as the treatment is now less aggressive and expensive [1].

NIFTP has been acclaimed as a “practice changer” in endocrine pathology [4]. A spectrum of adjustments in the pathological practice involves surgical pathology, cytopathology, and molecular pathology. A thorough sampling and evaluation of surgical specimens with strict adherence to the diagnostic criteria are mandatory to sign out NIFTP [1]. The preoperative thyroid fine-needle aspiration (FNA) cytology has limited utility in diagnosing NIFTP. As such, refinement of cytologic criteria to accommodate NIFTP in the lower-risk diagnostic categories has been recently proposed [5]. Labeling NIFTP as a non-cancer inevitably affected the major output of the cytologic reporting systems, a risk of malignancy (ROM). ROM in the reporting systems, for example, the Bethesda system for reporting thyroid cytopathology, is a crucial criterion for decision making. Low ROM suggests a conservative approach, whereas a high ROM indicates radical surgery. The ROM for each of the cytologic diagnostic categories was initially computed considering noninvasive eFV-PTC as a cancer [6]. However, the adoption of the term NIFTP should shift the malignancy risk and alter the management algorithm associated with each diagnostic category of the Bethesda system [7]. Moreover, the introduction of NIFTP as a non-malignant tumor changes the performance of molecular tests used in the preoperative triaging of thyroid nodules. For example, the adjustment to NIFTP significantly decreased the positive predictive value of the gene expression classifier Afirma and the mutation/fusion panel ThyroSeq v2 for indeterminate thyroid nodules [8–10].

Herein, we summarized the results of the NIFTP projects performed by the Working Group of Asian Thyroid FNA Cytology. The group was established in 2016 as a voluntary effort to promote communication among Asian pathologists and cytopathologists, share Asian practices, and conduct multi-institutional studies [11, 12]. Currently, this growing network of Asian thyroid pathologists includes representatives from China, India, Japan, South Korea, and several ASEAN countries.

## Incidence of NIFTP

The extent of all the changes related to the NIFTP reclassification apparently depends on the incidence of NIFTP in a certain population [2, 13]. In their seminal paper, Nikiforov et al. estimated that the reclassification of noninvasive eFV-PTC to NIFTP affects more than 45,000 patients worldwide annually [1]. These projections were based on the 18.6% rate of NIFTP among more than 3400 PTC cases retrospectively examined in one American and three Italian institutions [1]. Similar rates ranging from 15 to 28% were further reported in independent retrospective cohorts from the USA, Brazil, and Europe [13–16].

The Asian continent is a major contributor to the worldwide prevalence of thyroid cancer. The GLOBOCAN database estimated that 48% of all new thyroid cancer cases in 2012 were diagnosed in Asia [17]. The incidence of NIFTP in Asians has not been reported extensively as compared with the USA. To date, only several Koreans, one Japanese, one Chinese, and one Turkish institutions have published their original studies [18–25].

To evaluate the incidence of NIFTP in the Asian practice, we retrospectively collected the data from nine tertiary thyroid cancer centers representing six Asian countries [26]. Briefly, data on all patients with primary PTC were collected from the institutional surgical pathology databases. All the slides signed out as FV-PTC were reviewed and classified as infiltrative FV-PTC, invasive eFV-PTC, and NIFTP (i.e., noninvasive eFV-PTC). Only 206 NIFTPs of the 26,604 cases of PTC were identified (Table 1). Accordingly, the mean rate of FV-PTC (6.3%, range 2.2–9.8%) was much lower than that in the international multi-institutional cohort study conducted by Nikiforov et al. (37.9%) [1]. NIFTP accounted for 19.3% of all FV-PTCs in our series; however, a considerable variation among the nine institutions (0–82%) was observed.

**Table 1:** Low incidence of FV-PTC and NIFTP in Asian institutions based on the surgical pathology database

Institution	Period	PTC	All FV-PTC		Infiltrative FV-PTC		Invasive eFV-PTC		NIFTP	
		*n*	*n*	%	*n*	%	*n*	%	*n*	%
Japan 1	2007–2015	9727	271	2.8%	104	1.1%	117	1.2%	50	0.5%
Japan 2	2015	386	25	6.5%	5	1.3%	8	2.1%	12	3.1%
Korea 1	2008–2014	6269	240	3.8%	100	1.6%	45	0.7%	95	1.5%
Korea 2	2014	2111	171	8.1%	116	5.5%	50	2.4%	5	0.2%
China 1	2011–2016	5113	113	2.2%	77	1.5%	20	0.4%	16	0.3%
China 2	2012–2014	2190	187	8.5%	168	7.7%	13	0.6%	6	0.3%
Taiwan	2010–2011	380	22	5.8%	2	0.5%	2	0.5%	18	4.7%
Thailand	2013–2014	163	16	9.8%	7	4.3%	5	3.1%	4	2.5%
Vietnam	2016	265	25	9.4%	15	5.7%	10	3.8%	0	0%
Total		26,604	1070	4.0% (6.3%^a^)	594	2.2% (3.2%^a^)	270	1.0% (1.6%^a^)	206	0.8% (1.5%^a^)

Modified from Bychkov et al. [26]. PTC = all primary PTC including NIFTP; all FV-PTC = all PTC follicular variant, including infiltrative and encapsulated (both invasive and non-invasive); NIFTP = noninvasive eFV-PTC

*PTC* papillary thyroid carcinoma, *FV-PTC* follicular variant of PTC, *eFV-PTC* encapsulated follicular variant of PTC, *NIFTP* noninvasive follicular thyroid neoplasm with papillary-like nuclear features, *%* percent out of all PTC

^a^Average of particular tumor rates in nine series regardless of the number of patients

Next, an independent study was conducted to evaluate the impact of NIFTP on ROM in each Bethesda diagnostic category within a large cohort of thyroid nodules from Asian countries (*n* = 11,372), including 2044 nodules with surgical follow-up [27]. As a secondary output, a low incidence of NIFTP was validated in India, Japan, Korea, Taiwan, and Thailand (Table 2).

**Table 2:** Reported institutional incidence of NIFTP among patients with PTC (in chronological order)

Source	Country	City/institution	Database	Design	NIFTP	Raw data
Strickland et al. [13]	USA	Boston, MA (MGH)	Cytologic	Retro	28.0%	85/304
Nikiforov et al. [1]	Italy	Bologna	Surgical pathology	Retro	13.6%	71/523^a^
	Italy	Turin	Surgical pathology	Retro	25.0%	102/409^a^
	Italy	Pisa	Surgical pathology	Retro	18.7%	411/2197^a^
	USA	New York, NY (MSKCC)	Surgical pathology	Retro	18.8%	57/303^a^
Thompson [16]	USA	South California, 11 hospitals	Surgical pathology	Retro	25.0%	81/324
Rosario et al. [15]	Brazil	Belo Horizonte	Surgical pathology	Retro	15.0%	129/860^a^
Faquin et al. [14]	USA-Switzerland	multicenter (4 institutions): Boston, MA (MGH); Philadelphia, PA; Baltimore, MD; Lausanne	Cytologic	Retro	22.9%	173/756
Canberk et al. [18]	Turkey	Istanbul	Cytologic	Retro	27.6%	94/341
Godley et al. [28]	USA	Boston, MA (BMC)	Surgical pathology	Pro	9.1%	8/88
Pusztaszeri et al. [29]	Switzerland	Geneva	Surgical pathology	Retro	13.8%	86/625
Lee et al., 2017 [23]	Korea	Seoul (Konkuk University)	Surgical pathology	Retro	2.7%	21/769
Liu et al. [24]	China	Shandong	Surgical pathology	Retro	0.4%	20/5561
Saglietti et al. [30]	Switzerland	Lausanne	Surgical pathology	Retro	4.2%	9/216^b^
Song et al. [25]	Korea	Seoul (Chung-Ang University)	Surgical pathology	Retro	1.8%	26/1444
Cho et al. [19]	Korea	Seoul (Catholic University)	Surgical pathology	Retro	1.5%	95/6269
Layfield et al. [31]	USA	Columbia, MO	Cytologic	Retro	15.4%	16/104^a,b^
Golding et al. [32]	USA	Gainesville, FL	Surgical pathology	Retro	6.3%	50/796^b^
Bychkov et al. [26]	Japan	Kobe	Surgical pathology	Retro	0.5%	50/9727
	Japan	Fukuoka	Surgical pathology	Retro	3.1%	12/386
	Korea	Seoul (Catholic University)	Surgical pathology	Retro	1.5%	95/6269
	Korea	Seoul (Yonsei University)	Surgical pathology	Retro	0.2%	5/2111
	China	Shandong	Surgical pathology	Retro	0.3%	16/5113
	China	Wuxi	Surgical pathology	Retro	0.3%	6/2190
	Taiwan	Taipei	Surgical pathology	Retro	4.7%	18/380
	Thailand	Bangkok	Surgical pathology	Retro	2.5%	4/163
	Vietnam	Ho Chi Minh	Surgical pathology	Retro	0%	0/265
Singh et al. [33]	USA	Sacramento, CA	Surgical pathology	Retro	12.1%	21/174
Parente et al. [34]	Canada	Toronto	Surgical pathology	Retro	2.1%	102/4790
Hirokawa et al. [21]	Japan	Kobe	Surgical pathology	Retro	0.5%	54/10076
Jaconi et al. [35]	Italy	Monza	Cytologic	Pro	27.5%	14/51
Zhou et al. [36]	USA	Philadelphia, PA	Surgical pathology	Retro	4.8%	66/1368
	Italy	Rome	Surgical pathology	Retro	17.9%	69/386
	USA	Chicago, IL	Surgical pathology	Retro	2.8%	15/529
Kiernan et al. [37]	USA	Nashville, TN	Cytologic	Retro	5.3%	17/321^a^
Mainthia et al. [38]	USA	Boston, MA (MGH)	Surgical pathology	Retro	14.5%	194/1335
Li et al. [39]	USA	Ann Arbor, MI	Cytologic	Retro	6.7%	17/252
Bychkov et al. [27]	India	New Delhi	Cytologic	Retro	10.2%	15/147^b^
	Japan	Fukuoka	Cytologic	Retro	4.0%	9/223^b^
	Korea	Seoul (Catholic University)	Cytologic	Retro	3.4%	6/178^b^
	Korea	Seoul (Yonsei University)	Cytologic	Retro	2.4%	6/248^b^
	Taiwan	Taipei	Cytologic	Retro	6.1%	11/180^b^
	Thailand	Bangkok	Cytologic	Retro	8.9%	12/135^b^

*Retro* retrospective, *Pro* prospective

^a^Estimated, raw data were not provided

^b^Out of all thyroid malignancies

**Fig. 1:**
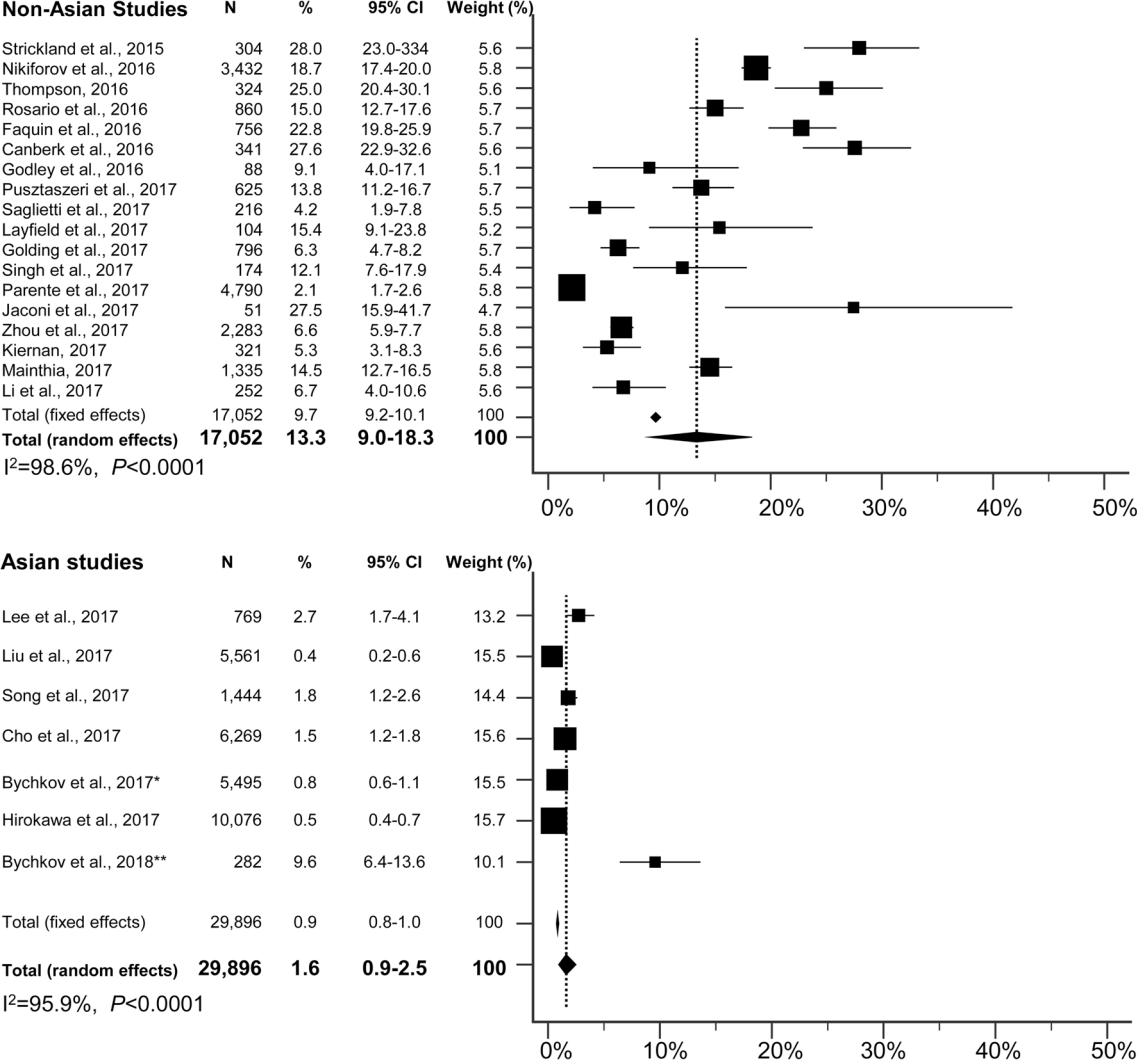
Forest plot of the NIFTP rate among PTC or thyroid malignancies in non-Asian and Asian series. The boxes indicate the point estimates for each study. The size of the box represents the weight given to each series. The whiskers indicate the 95% CI. Diamonds indicate the estimated average. The length of the diamond is the 95% CI for the combined average. *To avoid duplication, three institutions were excluded from the series of Bychkov et al. [26], because their cohorts were further expanded and reported in the original single-institution studies (Cho et al. [19], Liu et al. [24], and Hirokawa et al. [21]). **To avoid overlap, only data from India and Thailand are represented, because other cohorts were partially reported by Bychkov et al. [26]

We further collected the data from the available publications with reported institutional incidence of NIFTP (Table 2). To date, a total of 25 studies have already been conducted in America, Europe, and Asia [1, 13–16, 18, 19, 21, 23–39]. A meta-analysis was performed to estimate the overall prevalence of NIFTP among PTCs or thyroid malignancies in different countries (Fig. 1). We found an average rate of NIFTP was 9.1% (95% confidence interval [CI] = 6.0 to 12.7) of all PTCs worldwide. Asian studies reported a lower rate of NIFTP (1.6%, 95% CI = 0.9 to 2.5) compared with non-Asian countries (13.3%, 95% CI = 9.0 to 18.3; *p* < 0.001, chi-squared test). A study from Istanbul, Turkey, was categorized as the non-Asian group because Turkey is not a purely Asian country based on geography. Moreover, the Western publications, which were released immediately after proposing the NIFTP, reported that the rate of NIFTP (mean 20.9%, 95% CI = 14.4 to 27.4) was two times higher than those studied in 2017 (mean 10.4%, 95% CI = 5.5 to 15.3). This finding can be explained by the ongoing ramifications in the diagnostic criteria of NIFTP; for example, more strict requirements regarding the nuclear score are now recommended to avoid the overdiagnosis of NIFTP [19, 34].

Several factors related to data collection may influence the NIFTP rate. Among the important factors are the *prospective and retrospective designs*. In order to diagnose NIFTP, the adequate sampling must be done on the entire tumor capsule [1]. However, it was not previously considered as a standard method. Hence, some cases retrospectively reclassified from noninvasive eFV-PTC to NIFTP may not fit the NIFTP criteria, which could lead to the overestimation of the institutional NIFTP rate. To date, almost all the publications were done in a retrospective fashion, which is not surprising considering that the introduction of NIFTP was done in early 2016.

Another issue related to data collection is the *primary source of search*, which could be surgical or FNA database. Data on patients with NIFTP collected from the institutional FNA database require that FNA cytology and surgical resection should be performed at the same medical center, which often leads to the drop out of referral cases. Similar bias can originate from the database of single institution compared to the database of comprehensive health system that can accurately track the patients. In our experience, data on patients with NIFTP collected from the surgical database yielded a lower incidence of NIFTP as compared with the data collected from the FNA database; however, the coverage of the cytologic database was 1.5–2 times lower (e.g., 4.7% or 18/380 vs. 6.1% or 11/180 in Taiwan) [26, 27]. Hence, a primary search of NIFTP via surgical pathology database provides more precise incidence, while the studies reported the NIFTP rate based on a primary search via FNA database could likely overestimate a true incidence of this tumor, at least in our settings.

**Fig. 2:**
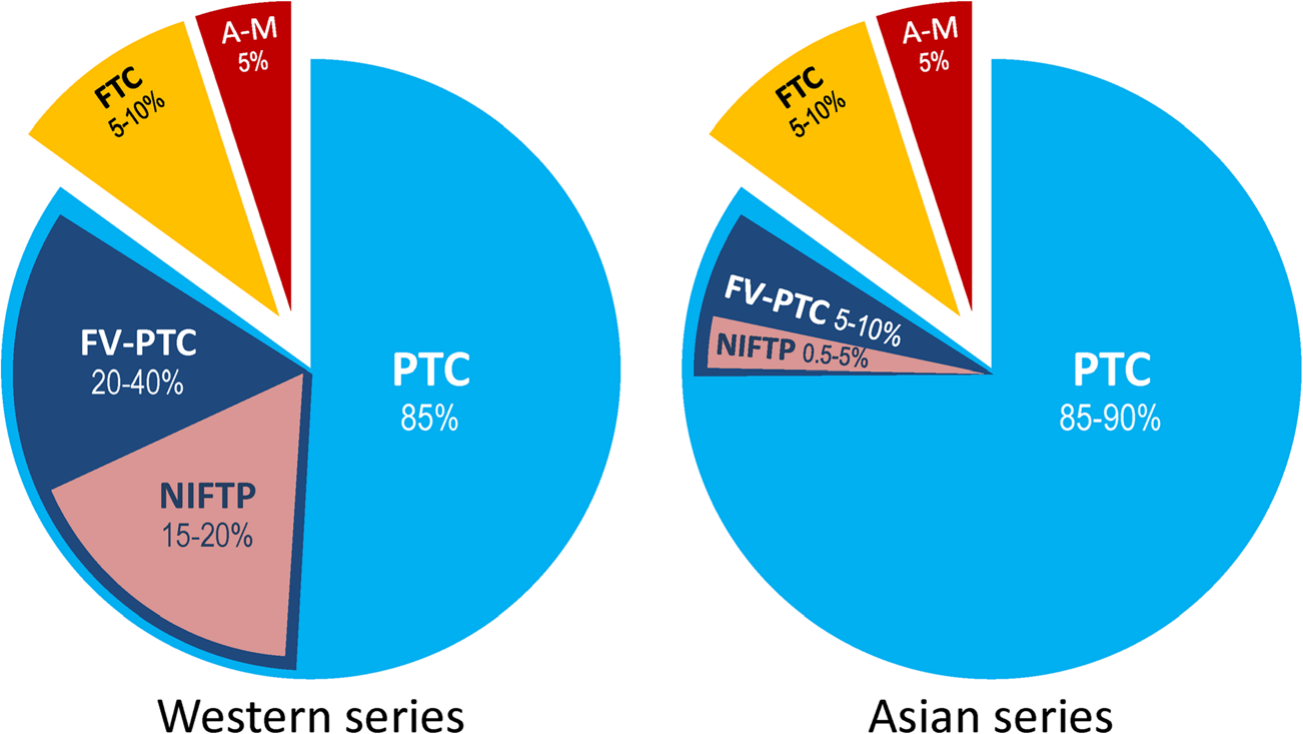
Incidence of FV-PTC and NIFTP in Western and Asian practice

The following are the *additional factors* affecting the incidence of NIFTP: the inclusion or exclusion of incidental microcarcinomas in the total number of cancers and calculation of NIFTP rate based on the number of all thyroid malignancies or only PTC cases. These variations in the study design are important but not critical because PTC is the predominant type of thyroid cancer accounting for 85–90% of all thyroid malignancies [40]. As shown in Table 2, there is a wide variation in the study design, which implies that the accurate rate of NIFTP worldwide needs to be established. Nevertheless, our preliminary estimates suggest that the initial rate of NIFTP worldwide (18.8% of all PTC) can be overestimated and that the incidence of NIFTP in the Asian population is much lower than that in the non-Asian series. The latter fact also applies to the entire group of FV-PTC (Fig. 2).

## Different Rates of NIFTP in Western and Asian Practice

Reasons for discrepancies between the Western and Asian series are multifactorial. A possible *difference in the histological interpretation of follicular-patterned thyroid lesions* can be one of the major causes. Despite the well-established diagnostic criteria, a high rate of disagreement regarding the evaluation of PTC nuclei and other features of FV-PTC occurred among expert pathologists [41–43]. The low agreement was reported between American and Japanese experts; in particular, Japanese pathologists more often favored benign diagnoses over malignant [42]. More recent reports showed that Korean pathologists have a higher threshold to recognize PTC nuclei than their Western colleagues [44]. In fact, this threshold continued to decrease among American pathologists in the last 10–15 years, which resulted in the growing incidence rate of FV-PTC, on the one hand, and decrease of follicular adenomas, on the other [45].

The study on the incidence of NIFTP in Asian countries (Table 1) reported a significant variation on the NIFTP rate among institutions (*p* < 0.001, chi-square test of independence) [26]. Thus, this prompted us to address the potential interobserver variability to evaluate the PTC nuclei, a major diagnostic characteristic of NIFTP and FV-PTC. A group of eight endocrine pathologists from Asian countries were recruited in this study (Z. Liu, unpublished data). These pathologists were informed about the diagnostic criteria, but no comprehensive training was given. A total of 30 original cases of NIFTP were reviewed using virtual slides and a nuclear scoring method (scores 1–3) as proposed by Nikiforov et al. [1]. The observers had a substantial agreement to reach an interpretation on the presence or absence of nuclear features in order to diagnose NIFTP (score 0–1 vs. 2–3) (kappa value 0.45). The interobserver agreement for diagnosing score 2–3 was generally moderate (kappa value 0.21). As regards the specific nuclear characteristics, the observers had a good agreement on the chromatin features, moderate for membrane irregularities, and fair for nuclear size and shape. These findings suggest that an interobserver variation exists among Asian pathologists when evaluating the microscopic features of PTC nuclei. This can be well illustrated by the case of two centers from the same city (Seoul, Korea), showing different rates of eFV-PTC and NIFTP (Table 1) which are linked to the distinct appreciation of histological features by the local pathologists.

As it was shown above with a time-dependent trend of lowering NIFTP prevalence since 2016, a strict adherence to the diagnostic inclusion and exclusion criteria is important to avoid overestimation of NIFTP rate. Korean pathologists promoted “no papillae cutoff” to correctly diagnose NIFTP [19]. When the same strict criteria were recently applied in the Canadian series, the incidence of NIFTP was 2.1%, which is very similar to the Asian estimates [34]. This implies that uniform interpretation of not only PTC nuclear features but also other histological criteria of NIFTP are necessary to consider for critical evaluation of NIFTP rate.

Another histological entity rarely recognized in the modern practice, which can be potentially misinterpreted as NIFTP, is a well-differentiated tumor of uncertain malignant potential (WDT-UMP). Before the introduction of NIFTP, WDT-UMP along with follicular tumor of uncertain malignant potential (FT-UMP) was a diagnostic resort for borderline thyroid tumors [46]. However, the use of a confusing terminology in the title inhibited the pathologists and clinicians from adopting this entity. Nevertheless, WDT-UMP was included in the last edition of the World Health Organization classification of thyroid tumors [40] and, despite its rarity, was diagnosed and signed out in the Asian institutions [47]. Most of American pathologists avoided the use of WDT-UMP and FT-UMP in their daily practice.

We speculate that the different appreciation on the microscopic features of FV-PTC, especially higher thresholds in evaluating PTC nuclei, can be a clue for understanding the different rates between Asian and Western series. However, a variable histological interpretation is unable to explain a five- to tenfold difference. Another major reason is that the *geographic* (e.g., iodine content) *and ethnic* (genetic traits) *background* may influence the distinct mutation profile and molecular mechanisms of PTC in Asians [48–50]. The increased prevalence of *BRAF*^V600E^ mutation in Korean and Japanese patients renders a predominance of classic PTC over FV-PTC [48]. For example, according to the Korean National Cancer Information Center (www.cancer.go.kr), PTC and follicular carcinoma accounted for 95.9 and 1.7%, respectively, of all thyroid malignancies in 2014.

Another important contributing factor is the *different approach in the management of cytologically indeterminate thyroid nodules*, which are often represented by NIFTP [13, 14, 51, 52]. These patients usually require surgery in the Western practice, whereas active surveillance without surgical intervention is favored in Asia [2, 53–55]. As a result, non-progressing NIFTPs may remain unresected lifelong, which reduces the incidence in Asian series. On the other hand, the progression of NIFTP under surveillance may further evolve to invasive eFV-PTC or infiltrative FV-PTC. This speculation is supported by the finding that incidence of NIFTPs among all FV-PTCs is lower in Asian studies (19%) than in Western studies (61%) [9, 26].

In summary, we believe that the combination of several factors, including different diagnostic thresholds, different nature of PTCs, and different established clinical practices, may explain the lower incidence of NIFTP in the Asian practice.

## Impact of NIFTP on the Practice of Surgical Pathology

The pathologist must strictly follow the histologic criteria of NIFTP to make an accurate diagnosis of the tumor and exclude other follicular-patterned lesions, some of which are true cancers [1]. Questions regarding the number of sections that should be taken from the tumor frequently arise. The diagnostic criteria for NIFTP require a meticulous histologic examination of the entire tumor capsule and tumor interface including the adjacent tissues to rule out capsular or vascular invasion of the tumor. Although there is no consensus on how many sections of the internal tumor component should be taken, authors believe that all components of the tumor should be submitted for histologic examination to determine the presence of invasion, true papillae, mitoses, psammoma bodies, solid/trabecular/insular growth pattern, and tumor necrosis. Despite its importance, the entire tissue sampling may exact a costly toll on the resources of the surgical pathology laboratory.

The Working Group of Asian Thyroid FNA Cytology conducted a survey to identify the opinions and the changes in practices and the impact of NIFTP on surgical pathology from August 23, 2017 to September 20, 2017. We recruited thyroid pathologists from six Asian countries (China, India, Japan, Korea, Taiwan, and Thailand) to complete an online questionnaire via e-mail. Each pathologist represented a single institution. A total of 69 questionnaire responses were received, 59 of which provided a baseline data according to their practices (Table 3).

**Table 3:** Baseline characteristics of respondents and local training programs regarding NIFTP

	Total	Japan	China	Korea	Thailand	India	Taiwan
Respondents	59	20	11	13	8	6	1
Affiliation							
Academic	43	11	11	12	3	5	1
Other	16	9	0	1	5	1	0
Approximate number of thyroid surgical cases per year signed out by the participant							
Mean	455	242	956	580	265	165	800
Median	250	67	600	315	255	130	800
Did you have a local seminar about NIFTP introduction and diagnostic criteria?							
	30/59	5/20	9/11	8/13	4/8	4/6	0/1
Did you perform internal audit of PTC nuclear scoring at your department?							
	14/59	3/20	5/11	4/13	2/8	0/6	0/1

**Fig. 3:**
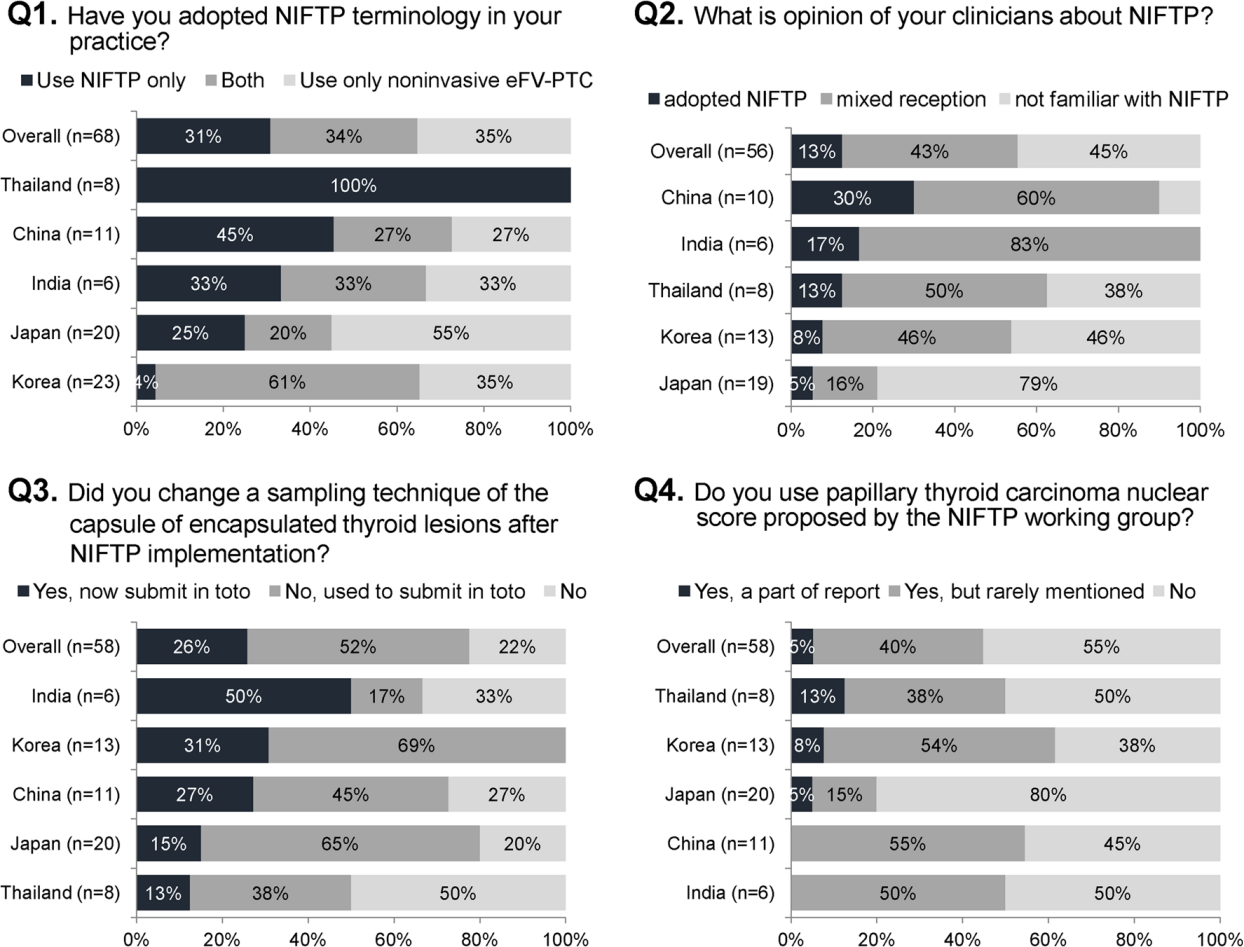
Responses of Asian pathologists to the NIFTP survey

Questionnaire results are summarized in Fig. 3. Taiwan was excluded as there was only one respondent. As regards the use of the term “NIFTP,” 31% of the respondents from five countries used NIFTP alone in their pathology reports; however, 34% pathologists used NIFTP in conjunction with noninvasive eFV-PTC to have a better understanding of the term. The remaining 35% still preferred to use the term “noninvasive eFV-PTC” instead of NIFTP. We also surveyed the opinion of clinicians about the NIFTP based on the perception of pathologists from the same multidisciplinary teams. Of the 56 hospitals, 13% have adopted the concept of NIFTP, 43% had mixed reception due to some uncertainty, and 45% have not started using the term NIFTP in the clinical practice. Interestingly, both pathologists and clinicians from several hospitals from China and India expressed their unfamiliarity with the term NIFTP.

The grossing technique for encapsulated thyroid tumor has been changed in 26% of the respondents after implementing the use of NIFTP. Of the 58 respondents, 52% used to submit the whole capsule before the NIFTP introduction, whereas 22% replied that they have certain limitations and can only submit a part of the capsule. The PTC nuclear score was routinely provided in pathology report by 5% of the respondents. In 40% of the respondents, the PTC nuclear score was used for diagnostic purposes but rarely mentioned in the report. It should be noted that the results of our survey conducted among the Asian countries are applicable to the checkpoint of “1.5 years after NIFTP proposal.” More progress in the adoption of the new terminology is anticipated, which need to be monitored in the next few years.

Encapsulated follicular-patterned thyroid tumors having questionable capsule or vascular invasion can be further divided into WDT-UMP and FT-UMP based on the presence or absence of PTC-type nuclear features. Of the 58 respondents, 26% separately used NIFTP and WDT-UMP depending on the extent of invasion, and another 26% preferred to differentiate all borderline thyroid tumors as either NIFTP or WDT-UMP/FT-UMP. However, 47% of the respondents avoided the use of these terms and still used benign (follicular adenoma or atypical adenoma) to cases with incomplete PTC-like nuclear features and malignant (noninvasive eFV-PTC) to cases with fully developed PTC nuclei. The original paper by Nikiforov et al. did not include NIFTP smaller than 1.0 cm [1]. Data regarding the sub-centimeter NIFTP are limited. In our survey, 90% applied the same histologic criteria for NIFTP regardless of the tumor size, and 10% replied that micro-NIFTP should be diagnosed as a follicular variant of papillary microcarcinoma.

When the prevalence of NIFTP among six Asian countries was roughly estimated based on the respondents’ recalls, the projected mean rate of NIFTP was 139 (0.6%) among all thyroid surgical cases over 1 year. Considering at least 20–40% prevalence rate of PTC among all thyroid surgical specimens, the NIFTP accounts for 1–3% of all PTC cases, which is in line with our previous findings (Table 1). A substantial geographic variability in the prevalence of NIFTP was observed, ranging from 0.04% in Japan to 3.2% in India.

Although the survey was only conducted to a few Asian countries, it showed different patterns of NIFTP adoption. The majority of respondents from China, India, Korea, and Thailand provided educational programs related to the introduction of the term NIFTP, such as local seminars (Table 3). As a result, most of the pathologists from these countries adopted the term NIFTP in their daily practice. On the other hand, 55% of the surveyed Japanese endocrine pathologists did not use the term NIFTP as a diagnosis and still favored to call such tumors as noninvasive eFV-PTC. Accordingly, a substantial amount of Japanese (79%) and Korean (46%) clinicians were not familiar with NIFTP. Thus, the pathologist is a key person in the thyroid multidisciplinary team who is responsible for the adoption and promotion of the NIFTP terminology through the provision of educational programs.

## Molecular Profile of NIFTP

Molecular studies have revealed that NIFTP, noninvasive eFV-PTC, and invasive eFV-PTC show similar molecular profiles and point to a common pathway of progression in encapsulated follicular-patterned tumors [1, 19, 56]. NIFTPs predominantly harbor point mutations in *RAS* and fusions involving the *PPARG* and *THADA* genes but lack *BRAF*^V600E^ [1]. However, recent Korean studies have reported the *BRAF*^V600E^-positive NIFTPs. Cho et al. reported that *BRAF*^V600E^ was detected in 10 (10%) of the 105 noninvasive eFV-PTC cases classified according to the inclusion of ≤ 1% papillae but was not found in the tumors in which the cutoff of 0% papillae (strict diagnostic criteria) was used [19]. These findings were comparable to another recent Korean study in which the *BRAF*^V600E^ was detected in 9 (12%) of the 74 noninvasive eFV-PTC cases but was not found in cases where any papillary structure was completely absent to define NIFTP [56]. Lee et al. reported the highest rate (29%, 6/21) of *BRAF*^V600E^ in Korean patients with NIFTP; however, the true mutation rate may have been overestimated as no clear details on histologic interpretation of NIFTP and inclusion/exclusion criteria were provided [23]. The non-V600E mutations in *BRAF* gene were found to be 4% (4/95) and 3% (2/74) in two Korean studies [19, 56]. *RAS* mutation rates in NIFTP were 47% (42/89), 49% (36/74), and 57% (12/21) as reported in three Korean studies [19, 23, 56]. Thus far, data regarding the comprehensive genotype of Asian NIFTPs, including fusions of *THADA* and *PPARG* as well as point mutations in *EIF1AX* and *TERT* promoter, are not yet available.

NIFTP was originally considered to have no lymph node metastasis and no *BRAF*^V600E^ mutation. However, the regional lymph node metastasis in NIFTP has been reported in Korean studies [19, 23, 56]. In our questionnaire, four Korean and one Indian respondent mentioned the presence of NIFTP with lymph node metastasis. Three Koreans and one Thai respondent mentioned that they have *BRAF*^V600E^-positive NIFTP. These results suggest that the current criteria for the histologic diagnosis of NIFTP may not be enough to exclude real PTC with malignant behavior and therefore need further refinement. Cho et al. suggested that the absence of papillae, rather than a 1% cutoff as initially proposed, was an important criterion when excluding *BRAF*^V600E^-positive tumors from NIFTP [19]. Results of our survey reiterate that the diagnostic criteria for NIFTP should be revised to become stricter with regard to exclusion criteria.

As NIFTP is by definition a borderline tumor, any tumors with cancer-specific mutations such as *BRAF*^V600E^, *RET*/*PTC* rearrangements, and *TERT* promoter mutations should not be diagnosed as NIFTP. Immunohistochemical staining or molecular testing for *BRAF*^V600E^ is recommended to differentiate true PTC from NIFTP in cases suspicious of microscopic morphology of conventional PTC such as florid nuclear features (nuclear score 3), presence of subtle papillae, or increased fibrosis. When immunostaining for mutant protein or molecular testing is not available, NIFTP should be diagnosed according to the strict diagnostic criteria through the meticulous histopathologic examination of the entire tumor.

## Cytopathology and Preoperative Diagnosis of NIFTP

Our group retrospectively analyzed the consecutive thyroid FNA samples from six institutions representing three geographically distinct parts of Asia, including eastern (Japan, South Korea, Taiwan), southern (India), and southeastern (Thailand) [27]. Of the 11,372 thyroid nodules aspirated at the six institutions, 2044 had available surgical follow-up (Table 2). The surgical pathology slides with a diagnosis of eFV-PTC were reviewed, and nodules were reclassified into invasive eFV-PTC and NIFTP. NIFTP was diagnosed in 59 cases, which constituted 2.9% of all excised thyroid nodules and 5.3% of malignant nodules [27]. The preoperative cytological diagnoses for NIFTP cases were non-diagnostic (10.2%), benign (18.6%), atypia of undetermined significance/follicular lesion of undetermined significance (AUS/FLUS, 22.0%), follicular neoplasm/suspicious for follicular neoplasm (FN/SFN, 32.2%), suspicious for malignancy (11.9%), and malignant (5.1%). Hence, two thirds of the NIFTP cases (66.1%) belonged to the indeterminate FNA categories.

This finding is consistent with that of the previous studies, which found that most of the NIFTP cases were clustered within the categories AUS/FLUS, FN/SFN, and suspicious for malignancy [13, 14, 18, 36, 37, 51, 52]. A considerable amount of NIFTP with non-diagnostic (14%), benign (15–17%), and malignant (9–13%) cytology has been reported recently [13, 14, 18]. In our study, 33.9% of NIFTP cases were classified as out of the indeterminate categories and occupied benign, malignant, and non-diagnostic groups, implying that this tumor can be preceded by any of the Bethesda categories.

**Fig. 4:**
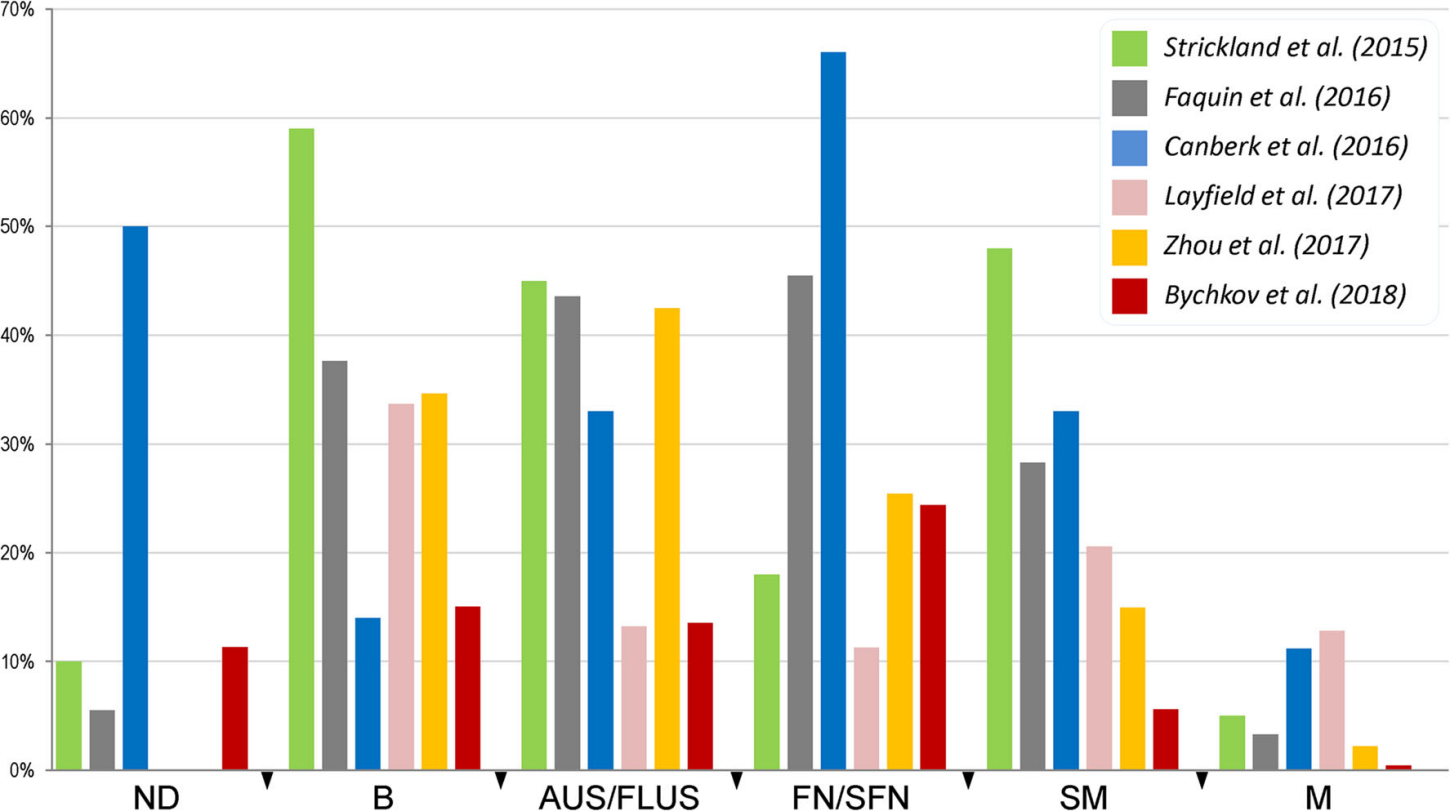
The impact of NIFTP on the relative decrease in ROM for the Bethesda diagnostic categories. ND non-diagnostic, B benign, AUS/FLUS atypia of undetermined significance/follicular lesion of undetermined significance, FN/SFN follicular neoplasm/suspicious for follicular neoplasm, SM suspicious for malignancy, M malignant

A recent meta-analysis based on four series showed that reclassification of noninvasive eFV-PTC to non-malignant NIFTP significantly affected ROM for indeterminate diagnostic categories [31]. In our multi-institutional cohort, the greatest relative decrease in ROM (24.4%) was observed for FN/SFN [27]. None of the other diagnostic categories, including AUS/FLUS and suspicious for malignancy, showed a relative ROM reduction of more than 20%. A comparison of the findings between our and previous studies [13, 14, 18, 27, 31, 36] is shown in Fig. 4. The low impact of NIFTP on the absolute and relative decrease in ROM in each of the Bethesda diagnostic category in our cohort compared with the Western series is directly attributed to the low incidence of NIFTP.

The pathological diagnosis of NIFTP requires evaluation of the capsule and therefore can be rendered on surgical samples only. However, some authors reported that the preoperative diagnosis of NIFTP can be suggested on the thyroid FNA [29, 51, 52, 57]. In our series (59 NIFTP vs. 48 invasive eFV-PTC), NIFTP exhibited a left-sided shift toward benign based on the Bethesda category scale, whereas the invasive eFV-PTC had a right-sided shift toward malignant categories [27]. This fits to the stepwise concept of the development of follicular-patterned thyroid tumors [1, 4]; however, definite distinctive cytological features were not established. A Korean group proposed that NIFTP is better triaged using core-needle biopsy instead of FNA [20]. It is likely that not a single marker or technique, but a combination of sonographic and cytologic findings potentially added by molecular tests, may provide the best opportunity to establish a diagnostic algorithm for the preoperative differentiation of NIFTP and true thyroid malignancies [20, 22, 58].

## Handling NIFTP in the Asian Practice

The term NIFTP meant that the tumor is not malignant but a borderline neoplasm. When the histologic diagnosis of NIFTP is rendered in patients undergoing thyroid lobectomy, no further treatment, such as an additional surgery and radioactive iodine ablation, is necessary [1, 2]. Patients will bene fit from the reduction of the harms of potential overtreatment, psychological burden of cancer diagnosis, complications possibly caused by further treatment, and medical expense. Nevertheless, most of the authorities consider NIFTP as a surgical disease, which requires resection for diagnostic purpose and to prevent progression to invasive phenotype [1, 2].

An alternative approach to borderline thyroid tumors, including NIFTP, was developed by Japanese institutions [53]. In general, it is applicable to the thyroid nodules with preoperative diagnosis of FN/SFN, which is largely represented by NIFTP. According to the Japan Thyroid Association clinical guidelines, diagnostic surgery is not indicated for patients with FN/SFN nodules when patients have benign clinical findings, and patients with FN/SFN nodules are actively monitored until any suspicious findings appear [53–55, 57]. As a result, this conservative clinical management for patients with cytological FN/SFN nodules establishes a low resection rate for this cytologic category compared with the Western practice. Moreover, it contributes to the higher ROM for surgically treated FN/SFN nodules with suspicious clinical and imaging findings as reported by several Asian studies [54, 55]. Consequently, in patients who underwent surgery after a strict risk stratification with ultrasound and FNA cytology, the proportions of larger size, advanced stage, and aggressive histology carcinomas increased; on the other hand, a fraction of small early-stage cancer, low-risk cancer, and borderline neoplasm decreased in addition to high ROM in all resected nodules (50–80%), which is a common observation in Asian series [55]. The pioneering clinical strategy of active surveillance successfully reduced the invasive diagnostic surgeries of patients with borderline thyroid tumors (NIFTP, WDT-UMP, and FT-UMP).

## Conclusion

Recent Asian studies provided additional knowledge for a better understanding of NIFTP. Our original data and the results of a meta-analysis suggested that the initial worldwide rate of NIFTP (18.8% of all PTC) could likely be overestimated and that the incidences of NIFTP and FV-PTC in the Asian population is much lower than those in non-Asian series. Such difference is contributed by various perceptions of histological diagnostic thresholds, different nature of PTC, and different approaches in the management of thyroid nodules. A concept of active surveillance for indeterminate nodules and NIFTP, largely represented in indeterminate cytologic categories, promoted by Japanese institutions, established a new paradigm to reduce the overtreatment of these patients. The lower prevalence of NIFTP in the Asian series indicates a low impact on the ROM in cytopathology and may predict a low impact on the performance of commercial molecular tests. Several Korean studies addressed the issue of *BRAF* mutation in NIFTP, which prompted the current refinement of the diagnostic criteria for NIFTP. Finally, our survey found that the term NIFTP has not been widely adopted in the Asian practice. Endocrine pathologists must be further motivated to promote the new entity through the provision of educational programs.

## References

[1] Nikiforov YE, Seethala RR, Tallini G, Baloch ZW, Basolo F, Thompson LD, Barletta JA, Wenig BM, Al Ghuzlan A, Kakudo K, Giordano TJ, Alves VA, Khanafshar E, Asa SL, El-Naggar AK, Gooding WE, Hodak SP, Lloyd RV, Maytal G, Mete O, Nikiforova MN, Nose V, Papotti M, Poller DN, Sadow PM, Tischler AS, Tuttle RM, Wall KB, LiVolsi VA, Randolph GW, Ghossein RA (2016). Nomenclature revision for encapsulated follicular variant of papillary thyroid carcinoma: A paradigm shift to reduce overtreatment of indolent tumors. JAMA Oncol.

[2] Haugen BR, Sawka AM, Alexander EK, Bible KC, Caturegli P, Doherty GM, Mandel SJ, Morris JC, Nassar A, Pacini F, Schlumberger M, Schuff K, Sherman SI, Somerset H, Sosa JA, Steward DL, Wartofsky L, Williams MD (2017). American Thyroid Association Guidelines on the Management of Thyroid Nodules and Differentiated Thyroid Cancer Task Force review and recommendation on the proposed renaming of encapsulated follicular variant papillary thyroid carcinoma without invasion to noninvasive follicular thyroid neoplasm with papillary-like nuclear features. Thyroid.

[3] Baloch ZW, Harrell RM, Brett EM, Randolph G, Garber JR (2017). American Association of Clinical Endocrinologists and American College of Endocrinology disease state commentary: Managing thyroid tumors diagnosed as noninvasive follicular thyroid neoplasm with papillary-like nuclear features. Endocr Pract.

[4] LiVolsi VA, Baloch ZW (2017). Coming to terms with diagnosis "non-invasive follicular neoplasm with papillary like nuclear features (NIFTP)": Practice changer in endocrine pathology. J Basic Clin Med.

[5] Pusztaszeri M, Rossi ED, Auger M, Baloch Z, Bishop J, Bongiovanni M, Chandra A, Cochand-Priollet B, Fadda G, Hirokawa M, Hong S, Kakudo K, Krane JF, Nayar R, Parangi S, Schmitt F, Faquin WC (2016). The Bethesda System for Reporting Thyroid Cytopathology: Proposed modifications and updates for the second edition from an international panel. Acta Cytol.

[6] Cibas ES, Ali SZ (2009). The Bethesda System for Reporting Thyroid Cytopathology. Am J Clin Pathol.

[7] Baloch ZW, Seethala RR, Faquin WC, Papotti MG, Basolo F, Fadda G, Randolph GW, Hodak SP, Nikiforov YE, Mandel SJ (2016). Noninvasive follicular thyroid neoplasm with papillary-like nuclear features (NIFTP): A changing paradigm in thyroid surgical pathology and implications for thyroid cytopathology. Cancer Cytopathol.

[8] Hang JF, Westra WH, Cooper DS, Ali SZ (2017). The impact of noninvasive follicular thyroid neoplasm with papillary-like nuclear features on the performance of the Afirma gene expression classifier. Cancer Cytopathol.

[9] Sahli ZT, Umbricht CB, Schneider EB, Zeiger MA (2017). Thyroid nodule diagnostic markers in the face of the new diagnosis, NIFT-P: Time for a reset?. Thyroid.

[10] Valderrabano P, Khazai L, Leon ME, Thompson ZJ, Ma Z, Chung CH, Hallanger-Johnson JE, Otto KJ, Rogers KD, Centeno BA, McIver B (2017). Evaluation of ThyroSeq v2 performance in thyroid nodules with indeterminate cytology. Endocr Relat Cancer.

[11] Bychkov A, Kakudo K, Hong SW (2017). Current thyroid FNA practices in Asia – A missing voice. J Pathol Transl Med.

[12] Jung CK, Hong SW, Bychkov A, Kakudo K (2017). The use of fine-needle aspiration (FNA) cytology in patients with thyroid nodules in Asia: A brief overview of studies from the Working Group of Asian Thyroid FNA Cytology. J Pathol Transl Med.

[13] Strickland KC, Howitt BE, Marqusee E, Alexander EK, Cibas ES, Krane JF, Barletta JA (2015). The impact of noninvasive follicular variant of papillary thyroid carcinoma on rates of malignancy for fine-needle aspiration diagnostic categories. Thyroid.

[14] Faquin WC, Wong LQ, Afrogheh AH, Ali SZ, Bishop JA, Bongiovanni M, Pusztaszeri MP, VandenBussche CJ, Gourmaud J, Vaickus LJ, Baloch ZW (2016). Impact of reclassifying noninvasive follicular variant of papillary thyroid carcinoma on the risk of malignancy in The Bethesda System for Reporting Thyroid Cytopathology. Cancer Cytopathol.

[15] Rosario PW, Mourao GF, Nunes MB, Nunes MS, Calsolari MR (2016). Noninvasive follicular thyroid neoplasm with papillary-like nuclear features. Endocr Relat Cancer.

[16] Thompson LD (2016). Ninety-four cases of encapsulated follicular variant of papillary thyroid carcinoma: A name change to Noninvasive Follicular Thyroid Neoplasm with Papillary-like Nuclear Features would help prevent overtreatment. Mod Pathol.

[17] Ferlay J, Soerjomataram I, Ervik M, Dikshit R, Eser S, Mathers C, Rebelo M, Parkin DM, Forman D, Bray F (2013) GLOBOCAN 2012 v1.0, Cancer Incidence and Mortality Worldwide: IARC CancerBase No. 11. http://globocan.iarc.fr. Accessed October 18 2016

[18] Canberk S, Gunes P, Onenerk M, Erkan M, Kilinc E, Kocak Gursan N, Kilicoglu GZ (2016). New concept of the encapsulated follicular variant of papillary thyroid carcinoma and its impact on the Bethesda System for Reporting Thyroid Cytopathology: A single-institute experience. Acta Cytol.

[19] Cho U, Mete O, Kim MH, Bae JS, Jung CK (2017). Molecular correlates and rate of lymph node metastasis of non-invasive follicular thyroid neoplasm with papillary-like nuclear features and invasive follicular variant papillary thyroid carcinoma: the impact of rigid criteria to distinguish non-invasive follicular thyroid neoplasm with papillary-like nuclear features. Mod Pathol.

[20] Hahn SY, Shin JH, Lim HK, Jung SL, Oh YL, Choi IH, Jung CK (2017). Preoperative differentiation between noninvasive follicular thyroid neoplasm with papillary-like nuclear features (NIFTP) and non-NIFTP. Clin Endocrinol (Oxf).

[21] Hirokawa M, Higuchi M, Suzuki A, Hayashi T, Kuma S, Miyauchi A (2017) Noninvasive follicular thyroid neoplasm with papillary-like nuclear features: a single-institutional experience in Japan. Endocr J. doi:10.1507/endocrj.EJ17-0214 10.1507/endocrj.EJ17-0214 28904306

[22] Jeon MJ, Song DE, Jung CK, Kim WG, Kwon H, Lee YM, Sung TY, Yoon JH, Chung KW, Hong SJ, Baek JH, Lee JH, Kim TY, Shong YK, Kim WB (2016). Impact of reclassification on thyroid nodules with architectural atypia: From non-invasive encapsulated follicular variant papillary thyroid carcinomas to non-invasive follicular thyroid neoplasm with papillary-like nuclear features. PLoS One.

[23] Lee SE, Hwang TS, Choi YL, Kim WY, Han HS, Lim SD, Kim WS, Yoo YB, Kim SK (2017). Molecular profiling of papillary thyroid carcinoma in Korea with a high prevalence of BRAFV600E mutation. Thyroid.

[24] Liu Z, Song Y, Han B, Zhang X, Su P, Cui X (2017). Non-invasive follicular thyroid neoplasm with papillary-like nuclear features and the practice in Qilu Hospital of Shandong University, China. J Basic Clin Med.

[25] Song RY, Kang KH, Kim HS, Park SJ (2017). Significance of follicular variant of papillary thyroid carcinoma: Study from a thyroid cancer center. Int J Thyroidol.

[26] Bychkov A, Hirokawa M, Jung CK, Liu Z, Zhu Y, Hong SW, Satoh S, Lai CR, Huynh L, Kakudo K (2017). Low rate of noninvasive follicular thyroid neoplasm with papillary-like nuclear features in Asian practice. Thyroid.

[27] Bychkov A, Keelawat S, Agarwal S, Jain D, Jung CK, Hong SW, Lai CR, Satoh S, Kakudo K (2018) Impact of noninvasive follicular thyroid neoplasm with papillary-like nuclear features on the Bethesda system for reporting thyroid cytopathology: A multi-institutional study in five Asian countries. Pathology (in press) 10.1016/j.pathol.2017.11.088 29631726

[28] Godley FA, Toraldo G, Mcaneny D, Doherty GM, Lee SL (2016). Noninvasive encapsulated follicular thyroid neoplasm with papillary-like nuclear features (NIFTP): A single institution prospective analysis. Thyroid.

[29] Pusztaszeri MP, Triponez F, Meyer P, Sadowski SM (2017). Noninvasive follicular thyroid neoplasm with papillary-like nuclear features (NIFTP): Report of an institutional experience with 86 cases. J Basic Clin Med.

[30] Saglietti C, Bongiovanni M (2017). The value of cytological examination in the diagnosis of noninvasive thyroid neoplasm with papillary-like nuclear features (NIFTP). J Basic Clin Med.

[31] Layfield LJ, Baloch ZW, Esebua M, Kannuswamy R, Schmidt RL (2017). Impact of the reclassification of the non-invasive follicular variant of papillary carcinoma as benign on the malignancy risk of the Bethesda System for Reporting Thyroid Cytopathology: A meta-analysis study. Acta Cytol.

[32] Golding A, Shively D, Bimston DN, Harrell RM (2017). Noninvasive encapsulated follicular variant of papillary thyroid cancer: Clinical lessons from a community-based endocrine surgical practice. Int J Surg Oncol.

[33] Singh R, Avila J, Jo K, Nguyen KTK, Carrillo NR, Huang EC, Campbell MJ (2017). Patients with non-invasive follicular thyroid neoplasm with papillary-like nuclear features are unlikely to have malignant preoperative cytology. Ann Surg Oncol.

[34] Parente DN, Kluijfhout WP, Bongers PJ, Verzijl R, Devon KM, Rotstein LE, Goldstein DP, Asa SL, Mete O, Pasternak JD (2017) Clinical safety of renaming encapsulated follicular variant of papillary thyroid carcinoma: Is NIFTP truly benign? World J Surg. doi:10.1007/s00268-017-4182-5 10.1007/s00268-017-4182-5 28828746

[35] Jaconi M, Manzoni M, Pincelli AI, Giardini V, Scardilli M, Smith A, Fellegara G, Pagni F (2017). The impact of the non-invasive follicular thyroid neoplasm with papillary-like nuclear feature terminology in the routine diagnosis of thyroid tumours. Cytopathology.

[36] Zhou H, Baloch ZW, Nayar R, Bizzarro T, Fadda G, Adhikari-Guragain D, Hatem J, Larocca LM, Samolczyk J, Slade J, Rossi ED (2017) Noninvasive follicular thyroid neoplasm with papillary-like nuclear features (NIFTP): Implications for the risk of malignancy (ROM) in the Bethesda System for Reporting Thyroid Cytopathology (TBSRTC). Cancer Cytopathol. doi:10.1002/cncy.21926 10.1002/cncy.21926 28941185

[37] Kiernan CM, Weiss VL, Mehrad M, Ely K, Baregamian N, Solórzano CC (2017) New terminology-noninvasive follicular neoplasm with papillary-like nuclear features (NIFTP) and its effect on the rate of malignancy at a single institution. Surgery. doi:10.1016/j.surg.2017.04.041 10.1016/j.surg.2017.04.041 29126555

[38] Mainthia R, Wachtel H, Chen Y, Mort E, Parangi S, Sadow PM, Lubitz CC (2017) Evaluating the projected surgical impact of reclassifying noninvasive encapsulated follicular variant of papillary thyroid cancer as noninvasive follicular thyroid neoplasm with papillary-like nuclear features. Surgery. doi:10.1016/j.surg.2017.04.037 10.1016/j.surg.2017.04.037 PMC5736433 29146229

[39] Li W, Sciallis A, Lew M, Pang J, Jing X (2017) Implementing noninvasive follicular thyroid neoplasm with papillary-like nuclear features may potentially impact the risk of malignancy for thyroid nodules categorized as AUS/FLUS and FN/SFN. Diagn Cytopathol. doi:10.1002/dc.23866 10.1002/dc.23866 29193839

[40] Lloyd RV, Osamura RY, Klöppel GK, Rosai J (2017). WHO Classification of Tumours. Pathology and Genetics of Tumours of Endocrine Organs.

[41] Elsheikh TM, Asa SL, Chan JK, DeLellis RA, Heffess CS, LiVolsi VA, Wenig BM (2008). Interobserver and intraobserver variation among experts in the diagnosis of thyroid follicular lesions with borderline nuclear features of papillary carcinoma. Am J Clin Pathol.

[42] Hirokawa M, Carney JA, Goellner JR, DeLellis RA, Heffess CS, Katoh R, Tsujimoto M, Kakudo K (2002). Observer variation of encapsulated follicular lesions of the thyroid gland. Am J Surg Pathol.

[43] Lloyd RV, Erickson LA, Casey MB, Lam KY, Lohse CM, Asa SL, Chan JK, DeLellis RA, Harach HR, Kakudo K, LiVolsi VA, Rosai J, Sebo TJ, Sobrinho-Simoes M, Wenig BM, Lae ME (2004). Observer variation in the diagnosis of follicular variant of papillary thyroid carcinoma. Am J Surg Pathol.

[44] Jung CK, Kim C (2017). Effect of lowering the diagnostic threshold for encapsulated follicular variant of papillary thyroid carcinoma on the prevalence of non-invasive follicular thyroid neoplasm with papillary-like nuclear features: A single-institution experience in Korea. J Basic Clin Med.

[45] Mehrzad R, Nishino M, Connolly J, Wang H, Mowschenson P, Hasselgren PO (2016). The relationship between the follicular variant of papillary thyroid cancer and follicular adenomas. Surgery.

[46] Williams ED (2000). Guest editorial: Two proposals regarding the terminology of thyroid tumors. Int J Surg Pathol.

[47] Liu Z, Zhou G, Nakamura M, Koike E, Li Y, Ozaki T, Mori I, Taniguchi E, Kakudo K (2011). Encapsulated follicular thyroid tumor with equivocal nuclear changes, so-called well-differentiated tumor of uncertain malignant potential: a morphological, immunohistochemical, and molecular appraisal. Cancer Sci.

[48] Song YS, Lim JA, Park YJ (2015). Mutation profile of well-differentiated thyroid cancer in Asians. Endocrinol Metab (Seoul).

[49] Bychkov A (2017). Prevalence of BRAFV600E mutation in Asian patients with thyroid cancer. Malays J Pathol.

[50] Liang J, Cai W, Feng D, Teng H, Mao F, Jiang Y, Hu S, Li X, Zhang Y, Liu B, Sun ZS (2017) Genetic landscape of papillary thyroid carcinoma in the Chinese population. J Pathol. doi:10.1002/path.5005 10.1002/path.5005 29144541

[51] Howitt BE, Chang S, Eszlinger M, Paschke R, Drage MG, Krane JF, Barletta JA (2015). Fine-needle aspiration diagnoses of noninvasive follicular variant of papillary thyroid carcinoma. Am J Clin Pathol.

[52] Maletta F, Massa F, Torregrossa L, Duregon E, Casadei GP, Basolo F, Tallini G, Volante M, Nikiforov YE, Papotti M (2016). Cytological features of "noninvasive follicular thyroid neoplasm with papillary-like nuclear features" and their correlation with tumor histology. Hum Pathol.

[53] The Japan Thyroid Association (2013). Guidelines for Clinical Practice for the management of Thyroid Nodules in Japan.

[54] Kakudo K (2017) How to handle borderline/precursor thyroid tumors in management of patients with thyroid nodules. Gland Surg. doi:10.21037/gs.2017.08.02 10.21037/gs.2017.08.02 PMC6107600 30175059

[55] Kakudo K, Higuchi M, Hirokawa M, Satoh S, Jung CK, Bychkov A (2017). Thyroid FNA cytology in Asian practice – Active surveillance for indeterminate thyroid nodules reduces overtreatment of thyroid carcinoma. Cytopathology.

[56] Kim TH, Lee M, Kwon AY, Choe JH, Kim JH, Kim JS, Hahn SY, Shin JH, Chung MK, Son YI, Ki CS, Yim HS, Kim YL, Chung JH, Kim SW, Oh YL (2017) Molecular genotyping of noninvasive encapsulated follicular variant of papillary thyroid carcinoma. Histopathology. doi:10.1111/his.13401 10.1111/his.13401 28940583

[57] Kakudo K, Kameyama K, Miyauchi A, Nakamura H (2014). Introducing the reporting system for thyroid fine-needle aspiration cytology according to the new guidelines of the Japan Thyroid Association. Endocr J.

[58] Yang GCH, Fried KO, Scognamiglio T (2017). Sonographic and cytologic differences of NIFTP from infiltrative or invasive encapsulated follicular variant of papillary thyroid carcinoma: A Review of 179 Cases. Diagn Cytopathol.

